# Psychological Symptoms in Patients with Injury-Related Chronic Pain

**DOI:** 10.5402/2012/196069

**Published:** 2012-03-15

**Authors:** Britt-Marie Stålnacke

**Affiliations:** Department of Community Medicine and Rehabilitation, Umeå University Hospital, Umeå University, 901 85 Umeå, Sweden

## Abstract

*Background*. Chronic injury-related pain could be influenced by psychological symptoms such as depression, anxiety, and stress that also affect daily life. *Methods*. Patients with chronic pain caused by an injury (*n* = 86) aged 18–65 years referred to the Pain Rehabilitation Clinic at the Umeå University Hospital answered a set of questionnaires to assess pain intensity, depression, anxiety, posttraumatic stress, sleep disturbance, and fatigue. *Results*. A significantly higher proportion of women (47.5%) reported depression (Hospital Anxiety and Depression Scale (HAD)) than men (22.2%). In all patients anxiety (HAD) was reported by 39.5% and moderate/severe posttraumatic stress (Impact of Event Scale) by 30.2%. A majority reported sleep disturbance (84.9%) and fatigue (90.7%). Significant relationships were found between posttraumatic stress and depression and anxiety. *Conclusion*. These findings indicate the importance of assessing and treating psychological symptoms associated with chronic pain as the result of trauma.

## 1. Introduction

Chronic pain (more than three-month duration) [1] is an acute and/or intermittent pain that persists and seems to be related to a complex interaction of risk and maintaining factors. About 20% of the Swedish population suffers from chronic pain, and a majority of this pain is musculoskeletal pain. Chronic pain has become a major health problem due to the high number of people affected. Apart from individual suffering, chronic pain is the cause of many social and financial pressures on society [2].

The causes of musculoskeletal chronic pain vary and are in several patients unknown. Some of the most common causes reported are injuries related to traffic accidents, falls, or sports injuries. In western countries, whiplash as the result of traffic injuries has a high annual incidence with 1.0 to 3.2/1000 per year [3, 4]. Whiplash is not only the term of the injury, it also describes the mechanism of energy—acceleration being transferred to the neck,—that results in soft tissue injury/distortion of the neck [5].

Chronic injury-related pain may be influenced by different physical and emotional factors that also affect daily life. During the last decade, attention has been paid to psychological factors such as depressive symptoms, anxiety, and negatively coloured cognitions that could be associated with future impairments [6, 7].

Moreover, posttraumatic stress reactions might play an important role in the persistence of symptoms after injuries and chronic pain after traffic accidents. In patients with traffic injury-related problems, 10–30% of the patients suffer from severe post-traumatic stress symptoms and receive a post-traumatic stress disorder (PTSD) diagnosis [8]. Although the severity of post-traumatic stress decreases with time, it has been shown that about one-third of traffic victims still suffer from post-traumatic stress reactions with anxiety and avoidance behaviour up to six years after the accident [8].

It is largely unknown how depression, anxiety, and posttraumatic stress interact in patients with injury-related chronic pain long after injury. A previous pilot indicated relationships between pain and stress, depression, and anxiety in patients with chronic pain [9]. Since most patients with chronic pain report poor-quality sleep that may influence the perceived level of depression and pain [10], the present study aimed to investigate all these factors in patients with chronic pain after trauma and who were referred to specialist care. A second aim was to examine differences between men and women.

## 2. Methods

### 2.1. Subjects

Participating subjects were 86 patients, 59 women and 27 men, aged 18–65 (41.1 ± 10.3) years, with chronic pain caused by an injury and referred from regional general practitioners to the Pain Rehabilitation Clinic at the Umeå University Hospital (Umeå, Sweden) between October the 1st 2007 and September 30th 2008. Written, signed informed consent was obtained from all participants. The participants suffered from pain caused by falls (14.0%), whiplash injuries (44.4%), other traffic injuries (bicycle, motorcycle and noncar injuries except whiplash) (8.1%), horseback riding (8.1%), sports (1.2%), assaults (5.8%), and other injuries (such as work related, etc.) (18.4%). The time between injury and assessment was more than one year in all patients.

### 2.2. Assessments

Patients answered a set of questionnaires at the assessment in the clinic. Information about each participant's trauma history was collected from hospital records.

#### 2.2.1. The Visual Analogue Scale

Pain intensity was rated by the Visual Analogue Scale (VAS). The scale consists of a 100 mm straight line with defined end points (“no pain” and “worst pain imaginable”) on which the patients were asked to mark their experienced pain (results in mm). The VAS is considered to have a high degree of reliability and validity [11].

#### 2.2.2. HAD

The Hospital Anxiety and Depression Scale (HAD) is an instrument that measures anxiety and depression which is developed and validated on nonpsychiatric medical patients. The questionnaire comprises of 14 items divided into two parts, for rating of depression and anxiety. Each item has a 4-response category range between 0 and 3. The scale ranges between 0 and 21 for both depression and anxiety. According to Zigmond and Snaith the cutoff level for possible cases of anxiety disorder and depression is a score ≥8 on each subscale [12].

#### 2.2.3. The Impact of Event Scale

The Impact of Event Scale (IES) is a widely used self-report scale. It is a valid measure of posttraumatic stress reactions and has been suggested as a screening tool for posttraumatic stress disorder (PTSD). The IES comprises 15 statements: seven questions regarding intrusive symptoms and eight address avoidance symptoms. The patients answer the questionnaire regarding their symptoms during the last week. A total score can vary from 0 to 80 [13]. According to the subclassification by Kongsted et al. [14] the score was divided into the grades subclinical to mild 0–25, moderate to severe 26–75 stress reactions. A total score of 35 has been suggested as cutoff for the diagnosis PTSD.

#### 2.2.4. Sleep Disturbance and Fatigue

The symptoms sleep disturbance and fatigue were rated on the Rivermead Post-Concussion Symptoms Questionnaire [3]. Patients are asked to rate the extent to which these symptoms have been more of a problem over the previous 24 hours compared with the premorbid levels, using a rating scale with values 0–4.

### 2.3. Statistical Analyses

All statistical analysis was performed with SPSS, version 19.0. Data are reported as mean values ± standard deviations unless otherwise indicated. Comparisons of populations were made using the Mann-Whitney *U* test. Pearson's correlation coefficient was calculated for the analysis of bivariate correlations. The results of logistic regression analysis are presented as OR (odds ratio), which indicates an increased (OR > 1) or a decreased (OR < 1) likelihood of the event (coded as 1) occurring. The reliability of the OR is expressed as 95% confidence intervals (CI). Statistical significant level was set at 0.05. The study was approved by the ethics committee of Umeå University.

## 3. Results

### 3.1. Pain Intensity

Pain intensity on the VAS for all patients was 65.8 ± 20.2 mm. No statistical significant difference was found between women (65.5 ± 21.1 mm) and men (66.8 ± 18.2 mm).

### 3.2. Depression

Depression scores on the HAD for all patients were 6.9 ± 4.4 mm (women 7.4 ± 4.3, men 5.9 ± 3.9). A significantly higher proportion of women (47.5%) reported possible-probable depression (HAD score ≥ 8) in comparison with men (22.2%) (*P* = 0.038). A statistically significant correlation was found between depression on the HAD and IES total score (*r* = 0.333, *P* = 0.003). In a logistic regression analysis of HAD depression, patients with possible-probable depression were chosen as a dependent variable (1 = patients with score ≥ 8, 0 = patients with score < 8). For patients with a possible-probable depression statistically significant association was found for moderate-to-severe posttraumatic stress (IES ≥ 26) (*P* = 0.020, OR = 3.246, CI: 1.201–8.774).

### 3.3. Anxiety

Anxiety scores on the HAD for all patients were 6.9 ± 4.2, women 6.9 ± 4.5 and men 7.0 ± 3.9. The frequency of patients with possible-probable anxiety (HAD score ≥ 8) was 39.5%. No statistically significant difference between men (40.7%) and women (39.0%) was found. A statistically significant correlation was found between anxiety on the HAD and IES total score (*r* = 0.483, *P* < 0.001). In a logistic regression analysis of HAD anxiety, patients with possible-probable anxiety were chosen as a dependent variable and coded as a binary variable (1 = patients with score ≥ 8, 0 = patients with score <  8). A possible-probable anxiety was statistically significant associated with moderate-to-severe posttraumatic stress (IES ≥ 26) (*P* = 0.020, OR = 3.246, CI: 1.201–8.774).

### 3.4. Posttraumatic Stress

Total scores of the IES for all patients were 19.3 ± 15.0 and the scores for the subscales Avoidance 9.8 ± 9.1 and Intrusion 9.5 ± 7.3. Subclinical stress response was reported by 32.6%, mild stress by 37.2%, moderate stress by 22.1%, and severe stress was reported by 8.1%. No statistically significant difference between men and women was found with respect to total IES (men: 19.7 ± 13.3, women: 19.1 ± 15.8), Avoidance (men: 10.2 ± 8.8, women: 9.6 ± 9.3), and Intrusion (men: 9.5 ± 5.9, women: 9.5 ± 7.8). In a logistic regression analysis of posttraumatic stress, patients with moderate-to-severe posttraumatic stress were chosen as a dependent variable and coded as a binary variable (1 = patients with score ≥ 26, 0 = patients with score >  26). A statistically significant association was found between moderate-to-severe posttraumatic stress and age <  40 years (*P* = 0.004, OR = 1.088, CI: 1.027–1.152).

### 3.5. Sleep Disturbance and Fatigue

The frequency of patients that reported sleep disturbance was 84.9% and of fatigue 90.7%. The scores of sleep disturbance for all patients were 2.86 ± 1.20 and for fatigue 3.01 ± 1.08. No significant difference was found between men (sleep disturbance: 2.88 ± 1.07, fatigue: 2.67 ± 1.35) and women (sleep disturbance: 2.84 ± 1.25, fatigue: 3.18 ± 0.889).

## 4. Discussion

The present study shows that the levels of depression, anxiety, and stress were high in patients with chronic injury-related pain and that a majority reported sleep disturbance and fatigue. The frequency of depression was significantly higher in women than in men.

### 4.1. Injury Cause

As whiplash is reported as the most common traffic injury, [5] it was not surprising that most patients related their chronic pain condition to a previous whiplash trauma. Although neck pain [15] is the dominating complaint after whiplash, symptoms of depression are commonly reported both in the acute phase and later after the injury [6].

### 4.2. Pain

Pain intensity on the VAS was rated high in the present study in comparison with several other chronic pain studies and higher in comparison with previous studies from our clinic [9, 16]. This may reflect the complexity of patients referred to specialist care.

### 4.3. Depression, Anxiety, and Posttraumatic Stress

Among psychological consequences after trauma, depression is frequently reported but the level of depression is seldom classified. In the present study a possible-probable depression was found in nearly 50% of women. These findings are clearly higher in comparison with previous studies that focus on whiplash injuries long after injury. Although there was no difference between men and women on the HAD anxiety, the frequency of anxiety was high in all patients. More than 40% of the patients fulfilled the criteria for possible-probable anxiety. The anxiety scores were in accordance with previous results from our clinic [9, 16] and clearly higher than those in an uninjured Swedish population [17] and indicate that a considerable proportion suffer anxiety long after trauma and that the level of anxiety may persist.

Psychological problems may also include posttraumatic stress reactions. Despite the fact that posttraumatic stress decreases with time, the level of moderate-to-severe stress (30.2%) was clearly higher in the present study than previous studies that documented patients both in the acute phase and several years after whiplash injuries [18, 19]. In the light of previous research, these results might reflect that the severity grade of posttraumatic stress may affect the experience of psychosocial functioning both after whiplash and after other injuries. In the logistic regression analyses, relationships between both possible-probable depression and moderate-severe stress and possible-probable anxiety and moderate-severe stress were found. Although there are few studies of the psychological complications long after injuries, several authors have emphasized that psychological problems should be seen as a consequence of somatic complaints [20].

### 4.4. Sleep Disturbance and Fatigue

Patients with chronic pain often report sleep dysfunction, and sleep dysfunction can aggravate pain [21]. In the present study, the great majority of the patients reported sleep disturbance and fatigue. These proportions were even higher than in previous studies of sleep dysfunction in chronic pain patients. Although sleep alterations are considered an important risk factor for psychological dysfunction and mental illness, these symptoms are seldom assessed together with depression and anxiety in patients with injury-related chronic pain.

Some limitations of this study should be noted. In this study general practitioners referred the patients to a pain rehabilitation clinic because of injury-related chronic pain due to an accident that occurred more than one year before the referral. Thus the results represent a selected group of patients with chronic pain with severe consequences after the injury, yet the most severe cases are referred to specialist departments at university hospitals. The results with high scores on the instruments therefore seem reasonable. Although patients reported symptoms that they referred to as the result of a previous injury, there may be symptoms of other origins that occur during the time between the trauma and the assessment. These factors might have influenced the patients' results.

This study has implications for clinicians, because the results indicate the importance of assessment of depression, anxiety, stress, and sleep disturbance in patients with chronic pain after trauma in order to optimize treatment.

In conclusion, this study shows high frequencies of psychological symptoms and sleep disturbance together with high pain intensity after trauma. These aspects should be taken into consideration in the management of patients with injury-related chronic pain.
